# Emergence of learning and quality - using scientific social media facilitating a complex adaptive space in healthcare

**DOI:** 10.1108/JHOM-07-2024-0284

**Published:** 2025-04-16

**Authors:** Jonas Boström, Malin Heimer, Johan Lilja

**Affiliations:** Department of Quality Management and Mechanical Engineering, Mid Sweden University, Östersund, Sweden; Västernorrland County Association of Local Authorities, Harnosand, Sweden

**Keywords:** Quality, Emergence, Healthcare, Organizational learning, Complexity, Transformation

## Abstract

**Purpose:**

Organizations are currently challenged to learn and develop quality at an increasing speed, as well as to navigate rising levels of complexity. This calls for new approaches to facilitating learning and quality as phenomena emerging in interconnected complex ecosystems of stakeholders. This paper explores the possibilities of facilitating the emergence of learning and quality in transformation and complexity with the support of scientific social media.

**Design/methodology/approach:**

The paper is based on a qualitative research design. Using scientific social media [SSM] for reflection and dialogue with an action research approach, it allows individuals with specific roles/functions linked to a transformative process to reflect on strong emotional experiences and action-oriented assignments. This can be described as equipping the ecosystem with sensors to capture systemic obstacles and levers.

**Findings:**

As a result, a triad with three themes of action possibilities for facilitating emergence was identified, with the support of scientific social media: (1) creating a living arena for emergence; (2) learning for emergence and (3) leading for emergence in transformation.

**Research limitations/implications:**

Future research could benefit from using scientific social media and combined qualitative and quantitative data to study quality and learning as emerging phenomena. Practically, organizations could use SSM for health system transformation.

**Originality/value:**

This paper provides empirical insights and new innovative ways of conducting research when exploring complex transformational changes in healthcare and the emergence paradigm of quality management.

## Introduction

The health care system is currently facing massive transformational changes to address the needs of the ageing population and, consequently, increasing demands from society. Resources are already lacking to sustain quality in the healthcare system as originally designed (World Health Organization, 2021). With limited resources to provide what is necessary now, even higher demands are expected in the future to simultaneously develop and transform processes and the healthcare system itself. Technology and digitization are mentioned as crucial factors. However, organizations seem not to be prepared for or receptive to these complex challenges (Cottam, 2011; Nason, 2023).

An additional complicating factor is that society has become increasingly complex, where the value and alignment of existing methods and tools for continuous improvement and process efficiency have become limited (Palmberg-Broryd, 2021; Stacey and Griffin, 2006; Van Kemenade, 2022). Therefore, the healthcare system must also strengthen the ability to lead and transform in complexity (Alvesson and Cizinsky, 2018; Andersson, 2015; Eriksson *et al.*, 2016).

In Sweden, a transformational shift started around 2015, aiming for more person-centered and integrated care, later referred to as Nära Vård, which emerged from the Swedish Ministry of Health and Social Affairs report “Good and Close Care – Collaborative Care” (SOU, 2019, p. 29) (see Table 1). This report also identified challenges in regard to relying on traditional quality methods and tools for supporting this transformational shift. Transformations in general (Lukas *et al.*, 2007) are often highly complex, and for the specific shift towards Nära Vård, the Swedish Association of Local Authorities and Regions [SALAR] (2022) has made a similar assessment, stating that this is unknown territory for both the organizations involved and their employees. Hölscher *et al.* (2018) emphasize that processes to shape transitions and transformations are also deeply political, involving power struggles and value conflicts. These include innovation (e.g. institutional, social, technological, economic), collaboration, learning and knowledge integration. The movement towards Nära Vård could also be seen as a pressure “pushing the system into disequilibrium” (Uhl-Bien, 2021, p. 148).

**Table 1:** The intended focus movements to achieve more person-centered and integrated care in Sweden

From	To
Focus on treatment and illness	Focus on health promotion and prevention
The individual as a passive recipient	The individual as an active and engaged partner
Hospital-based care	Open care forms
Isolated healthcare interventions	Coordination based on the individual’s needs

**Source(s):** SALAR (2022), Authors’ own work

**Figure 1:**
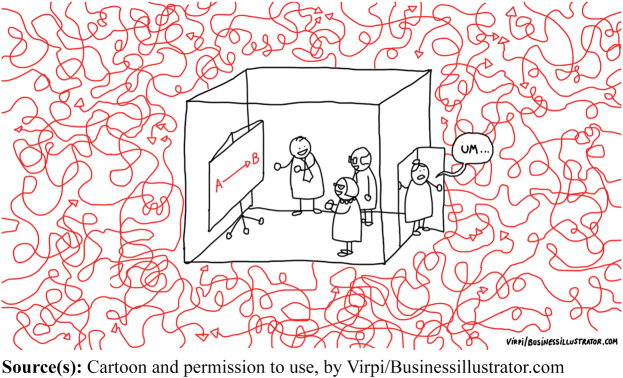
An illustration of the contrast between linear thinking and acting in a complex world

Batalden *et al.* (2015) argue that when complex phenomena are addressed, knowledge from various scientific fields is needed. In contrast, other studies within the healthcare context (Eriksson *et al.*, 2016; Gadolin and Andersson, 2017; Glouberman and Mintzberg, 2001; Kaplan *et al.*, 2010) show resistance to incorporating perspectives other than traditional medical ones when improving quality in healthcare. Another aggravating circumstance is noted in a report on Nära Vård (Rognes *et al.*, 2020) highlighting that the development of measures, indicators and currently available studies concerning healthcare are tailored to yesterday’s care and social services with linear thinking, not really suited for the complexity of today’s world, as illustrated in Figure 1.

From a quality perspective, this can be understood as acting from an outdated or unaligned paradigm of Quality Management (Van Kemenade, 2022; Van Kemenade and Hardjono, 2019). Twenty-first-century Total Quality Management (TQM) is proposed as needing a shift into the emergence paradigm that is better aligned with increasing complexity and uncertainty as well as the systemic challenges of our time.

Therefore, there is clearly a need to explore new practical ways of facilitating and following transformational change in complexity and to develop more knowledge about how to understand and facilitate the emergence of learning and quality in complexity. Nuño-Solinís (2017) specifically calls for this ability on an ecosystemic level in relation to achieving integrated care: “This transformation requires collaborative learning between cross-disciplinary teams and different care levels, and often between different healthcare organizations. It is essential to know and understand these dynamics and observe how they are influenced by professional cultures and the identities and cultures of the organizations” (p. 4).

Similarly, research on relational analysis (Gitell and Ali, 2021) shows that relational coordination is crucial to achieve increased quality, efficiency, learning and a good work environment. This implies that organizations must find ways of creating and holding space for cross-boundary roles, relational leadership and shared learning forums. Learning demands that time and the ability to reflect are available, both at the individual and organizational levels, to find improvements and/or innovative solutions (Kolb, 1984; Lackéus, 2021; Schön, 1991). Furthermore, Carroll and Edmondson’s (2002) research on leading organizational learning in healthcare highlights the need to facilitate connections between action and reflection.

In meeting those needs, technology such as scientific social media now enables new forms of real-time capturing and collective sharing and understanding of narratives and reflections within the ecosystem.

Using such scientific social media to invite streams of narratives into an arena or forum has the potential to provide conditions to create relationships and capture reflections on individuals' experiences of everyday working lives, aggregated at the group level. Different actions, experiences and conscious and unconscious choices could then make patterns, obstacles and leverage points visible, which the organization collectively can learn from and act upon. Thus, such technology could potentially open up a whole new set of possibilities to facilitate the emergence of learning and quality by relinking the parts and the whole, “making a system sense and see itself” (Scharmer, 2018, p. 17).

The purpose of this paper is therefore to explore the possibilities of facilitating the emergence of learning and quality in complexity with the support of scientific social media.

## Facilitating an adaptive space

Given the context of this paper, this chapter presents a brief review of systemic and transformational change, focusing on perspectives intended to be of specific importance for complexity, organizational adaptivity and facilitating change.

### Change in complexity

Nelson and Stolterman (2014) argued that the whole takes its emerging essence from its parts and that the relationship between the whole and its parts is inseparable. Along this line of reasoning, Stacey (2005) suggested that the organizational system cannot be described as an object that we can relate to; we should rather talk about it as the many interactions between people that take place all the time and the consequences of these interactions. Designing that system, then, is how we design the meetings of these people, making them visible to others and integrating the necessary resources. Snowden and Boone´s (2007) Cynefin framework gives an understanding of the challenges of leading in complexity and what is needed to cope with that. From that perspective a probe-sense-respond approach is favorable. Input on how to facilitate this can be found in social constructivist approaches. Hersted *et al.* (2020, p. 13) mention the importance of unfolding multiplicity and complexity and giving space for uncertainty, not seeking consensus. However, being in a state of disorder (Snowden and Boone, 2007) or staying in trouble (Juul-Søndergaard, 2018) is challenging.

**Figure 2:**
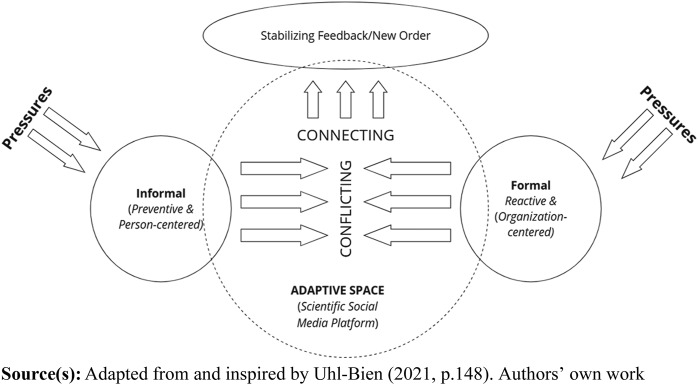
The adaptive process, showing how the transformation from the informal pressure from Nära Vård meets the formal pressure from traditional health care operations

Bringing in new and different perspectives to understand complex systems and facilitate change is highlighted as crucial (Gergen, 2023; Palmberg-Broryd, 2021; Roberts *et al.*, 2016; Stacey and Griffin, 2006). Embracing heterogeneity (Uhl-Bien, 2021) with different perspectives, stories and experiences broadens the possibility of finding new connections and coming to an understanding. Laloux (2020, p. 202) indicated that people are natural sensors and have the ability to notice when something is not working as it should or when an opportunity reveals itself. People cannot stop feeling; they feel something everywhere and all the time. Meeting and handling complexity challenges also demands organizational adaptability, especially when balancing the need to innovate and change in relation to ordinary operations, which could cause tensions and conflicts. Uhl-Bien and Arenas’ (2018) model on complex adaptive systems brings a theoretical framework to explore this tension. However, the potential conflict arising between the explorative and exploitative approaches is not always a barrier for change: it could also offer an opportunity for new things to emerge, as elaborated by Uhl-Bien and Arena (2018) in their notion of the adaptive space. The adaptive space then allows ideas from the informal (entrepreneurial) and formal (operational) systems to interact and connect in productive ways that generate emergence and a new adaptive order for a system. In this paper, this could be applied to the pressure of the transformational shift in healthcare (Figure 2) and where the adaptive space is closely related to the social media platform. Combining that with research on polarities (Johnson, 2014), the two pressures should be seen as interdependent pairs, meaning that positive energy could be a result if an organization seek to maximize the “upsides” with each polarity, and pay attention to early warnings from the “downsides”.

### Dialogue and learning

To facilitate the adaptive space (Uhl-Bien, 2021; Uhl-Bien and Arena, 2018), one could use different kinds of methods and tools. For example group model building [GMB], intending to engage stakeholders to collectively consider causes of complex problems (Gerritsen *et al*., 2020). However, for this study, the work around dialogic organizational development (Bushe and Marshak, 2014b, 2016a, b) and theories in relation to facilitation (Raelin, 2012; Schoobridge *et al.*, 2021), frames the way to collect and connect people, their perspectives, and experiences in relation to the pressure from a systemic transformation and the operational processes of daily work. “Leaders need to have an eye and ear for what people in the organization are saying, reading and writing about organizational dynamics” (Bushe and Marshak, 2016a, p. 41).

Uhl-Bien (2021) points to the facilitation skill leaders must have in creating space that helps people engage across differences and come up with adaptive solutions they can agree upon and move forward. This relates to Raelin (2012, p. 819), describing the facilitator as the “interlocutor” of dialogue, and with that the base for a mutually constructive dialogue between parties. When it comes to this study and this way of supporting transformational change, the study uses facilitators as an enabling mechanism (Schoobridge *et al.*, 2021). National Coalition for Dialogue and Deliberation [NCDD] principle #4, openness and learning, specifically promotes skilled and impartial facilitation, leading to a climate of conversation that creates conditions such as deep listening, a feeling of a safe space, diversity of perspectives and mutual exploration (Raelin, 2012).

Bushe and Marshak’s (2016b) dialogic OD mindset holds eight premises to support transformational change and states that a combination of three core processes must happen. Those three are emergence, happening when a disruption of the social order occurs and there is little chance of going back to a former state of mind (the established): this could be achieved by pushing the system close to chaos and prepare for self-organizing changes to emerge; narrative, or changing one or more core narratives in the organization: this requires another storyline to be told that has a big impact on what people think or their social agreements and generativity: this third transformation process suggests generative images that show another alternative to how we think or act. These images can take different forms, through words and or symbols. They also tend to make people want to act on them, being especially useful as triggers for fruitful dialogues and inquiry. Contrasting to this dialogic mindset is the diagnostic mindset (Table 2). However, it is not an either/or situation for organizations and/or leaders to choose between, but rather a combination depending on the context, challenge or problem (Hastings and Schwarz, 2022).

**Table 2:** Contrasting polar ideal types: diagnostic and dialogic mindsets

Diagnostic OD		Dialogic OD
Positivism Objective reality	ONTOLOGY	Interpretative Social Reality
Open systems	ORGANIZATIONS ARE	Dialogic Networks
Behavior and results	EMPHASIS ON	Discourse and Generativity
Planned Episodic More developmental	CHANGE IS	Emergent Continuous and iterative More transformational
Stay apart at the margins	CONSULTANTS	Are immersed with Part of
Hierarchical Start at top, work down	CHANGE PROCESSES	Heteriarchical Start anywhere, spread out

**Source(s):** Adapted from Bushe and Marshak (2014, p. 86), Authors’ own work

However, learning is highly dependent upon reflection, which, according to Kolb (1984) and others (Chan, 2023), is fundamental. Time for reflection must be available and facilitated to develop knowledge about what may need to be changed. Schön (1991) stressed that knowing is in our actions, and by that, often tacit: we know more than we express. Therefore, reflection becomes necessary to unfold this tacit knowing, as expressed by Tsoukas (2009, p. 1): “The engine of knowledge creation is articulation—a conscious process of making knowledge explicit”.

## Method

### Research design

This study is based on action research (Hersted *et al.*, 2020) and is supported by scientific social media (SSM) (Lackéus, 2014), which involves a mix of methods. This approach is particularly suitable when the focus is co-creation, learning and organizational development, as it facilitates the generation of new knowledge. The study adopts Reason and Bradbury’s (2008) definition of action research as “a participatory process concerned with developing practical knowing in the pursuit of worthwhile human purposes. It seeks to bring together action and reflection, theory and practice, in participation with others, in the pursuit of practical solutions to issues of pressing concern to people and, more generally, the flourishing of individual persons and their communities” (p. 4). This also relates to Hersted *et al.*’s (2020) findings and the view of ourselves as organizational change agents working from a future-oriented perspective.

As a longitudinal study with close relationships with the people involved and with the aim of considering the participants as collaborators/co-researchers (epistemic partners) and understanding different levels of complexity, this paper applies a para-ethnographic (Holmes and Marcus, 2008) approach.

### The context

The study used a virtual scientific social media platform created for systematic reflection to capture the stories and insights of individuals at the center of transformation, observing and reflecting on what members of the organization think and actually do in the transformation toward Nära Vård. To explore that, a specific region with a regional organization (health care) and municipalities (municipal care) was selected. The organizations decided to follow parts of the transformation process with this method, which can be considered a convenience sampling technique (Patton, 2015). The scope of the study involves data collected from 29 individuals in various roles (developers, coordinators, designers, project managers, etc.), linked through a specific declaration of intent between the region (organization) and connected municipalities, based on the national agenda of Nära Vård, which contains four focus movements (see Table 1) to sustain person-centered and integrated care. Each of these movements can also be understood as an intended transformation in the quality of care in Sweden with respect to perspectives, relationships and forms.

This context, with a transformational process as a phenomenon and complexity theories in mind, along with the iterative/cyclical mindset, opened up a unique space to explore how to facilitate the emergence of learning and quality.

### Data collection, analysis and the tool

The participants, hereafter referred to as co-researchers, were identified in collaboration with the organization, represented by a joint program office, to define roles that also provided a natural context for collective meta-reflections and analysis of the collected data. Written consent was obtained from the participants prior to the data collection. The selection aimed to represent the micro, meso and macro levels (Taillard *et al.*, 2016), with a particular emphasis on the meso level in this study.

**Figure 3:**
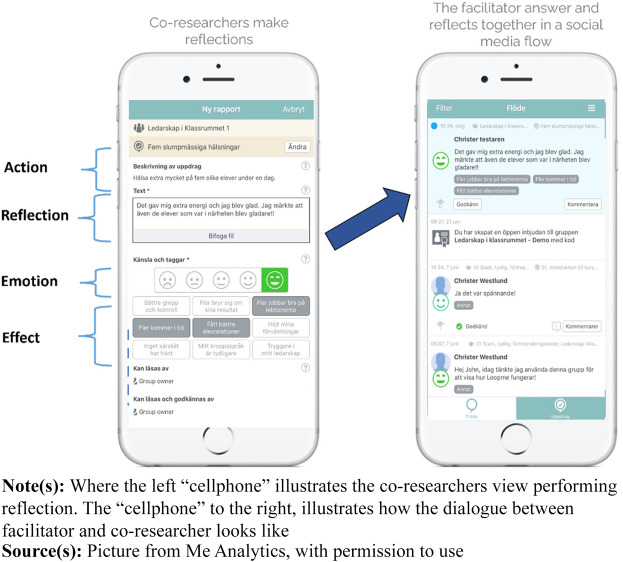
The social media tool (software application from LoopMe Group)

The study of SSM (Lackéus, 2014) was inspired by Kolb’s (1984) model of experiential learning. The SSM tool (Figure 3) has been developed from research on designed action sampling (Lackéus and Sävetun, 2023). Its design supports the collection of data from co-researchers in the form of free-text responses connected with a rating scale (self-assessment manikin) associated with various co-developed tags (effects). Reflections and learning occurred in four cyclical loops over one year (December 2022 to December 2023). Frequent separate meetings with the co-researchers, the steering group and a national Nära Vård group were held. The co-researchers were equipped with a smartphone (or a web-based program) application through which they received assignments (situations, actions or feelings) to reflect upon what was happening in the transformation process from their point of view and context in relation to Nära Vård. All the data were sampled in a master data file, which enabled both qualitative and quantitative data.

The co-researchers shared their individual reflections, which were responded to by the first author (facilitator) with follow-up/deepening questions. After each loop (assignment/action), the material was analyzed in a first step by the facilitators (first author and second author), following the process of reflexive thematic analysis (Braun and Clarke, 2022) throughout the process. Weekly reflection meetings (virtual) and monthly planning meetings (to plan the next step) were carried out. Notes were taken on a digital board (Miro) to identify what Patton (2015) described as patterns and meaningful stories from the qualitative data. The analytical journey (loop 1) first revealed early insights from an assigned reflection on what Nära Vård meant for them. These reflections were meta-analyzed with the co-researchers and management to determine the validity of the insights (Snowden, 2024), resulting in new questions and reflections to look for in their daily practice in relation to the transformation (loop 2).This time, the reflections revealed tensions and conflicts of interest that created new assignments (loop 3). From that, and at the end of the year, ten hypotheses were formulated that were objects for reflection (loop 4). For this paper, patterns and stories from the overall reflections involved a process of interpretation by the first two authors in relation to the four loops and the theoretical framework that ended with 277 codes (extracted datasets), abstracted to 31 subthemes, categorized down first to seven and finally three themes and one overall theme—enabling for a complex system to collectively sense, see and transform itself (see Table 2).

## Results and analysis

### Enabling for a complex system to collectively sense, see and transform itself

The collective results and analysis are presented as an identified triad of interconnected themes of action possibilities for facilitating the emergence of learning and quality in complexity with the support of scientific social media.

The first identified theme, creating a living arena for emergence, concerns the possibility of creating a different kind of shared space where the actors seem to prefer to be, become inspired and create joint awareness for change. The second theme, learning for emergence, stresses the possibility of using systematic learning, both vertically and horizontally, with listening and information as drivers. Finally, leading one another and not leading others in this landscape of complexity and the importance of being able to act consciously appeared as a third theme: leading for emergence in transformation.

These themes of action possibilities are described in more detail in Table 3, with interposed quotes from the co-researchers of the study.

**Table 3:** The final three themes of action possibilities, subthemes and summary of content

Enabling for a complex system to collectively sense, see and transform itself							
Themes	A living arena for emergence			Learning for emergence		Leading for emergence	
SUB-THEMES	Collective space	Participation	Catalysers	Basic needs	Systematics	Culture	Adaption
Summary	A neutral, illuminated and in-between space for togetherness A space that opens up for different and new perspectives A possibility to decouple traditional and formal structures			A need for systematic individual and collective reflection – documented An ability to use data and information A balance between thinking and doing An increased ability to listen to the organization		A need for a resilient and adaptive organization A change in leadership view – leading each other instead of leading others A change in how resources (people) are defined	

**Source(s):** Authors’ own work

## A living arena for emergence

The study stresses that facilitating the emergence of learning and quality is tightly connected to creating in-between spaces where no one is necessarily the formal manager in charge of others. This allows a joint responsibility and ownership to emerge. An illuminated arena is described by the co-researchers as necessary to obtain nourishment and commitment and to pursue tough issues together with others. The arena also creates the possibility of challenging prevailing norms, allowing the participants to question them together. However, the arena is not a place where the participants just sit passively; rather, they must act and contribute to a sense of the common and collective. That is, the arena should build energy: it does not consume energy. An arena that generates such energy is often built in a way such that many different perspectives can enter.

When you create arenas where employees from different organizations, principals, etc. meet and get the opportunity to build relationships, get to know each other, it facilitates meetings later where you have a common task to solve. Co-operation across borders is always easier and smoother if you know each other: it is more obvious to stand up for a “friend” than to help each other when you do not know each other or have a negative image of each other. (Co-researcher)

The reflections of the co-researchers further highlight the value of exploring the map and terrain together—that is the direction rather than the destination. This is considered a success factor in comparison to striving for separate goals. A common arena is mentioned as critical for creating action, for shared ownership and for holding a space where power and competence can be integrated, which is not otherwise done in the everyday operations of an organization. The reflections also signal that the other forums that exist today are not regarded as places where we jointly gain insight, discuss and co-create, but instead we guard our own interests.

[…] how important it is that we create arenas for “sharing the world” in an organization or system to enable shared responsibility and the development of trust. (Co-researcher)

I believe that employees will cooperate with others if they are only given the confidence to do so. (Co-researcher)

There are also difficulties finding arenas where interaction can be created between actors outside the traditional health care system. Here, the scientific social media platform might open up for new possibilities.

Even though ambition exists in the organization and commitment exists, we hinder the power of development by preventing the perspectives from meeting. There are so many untapped resources that can be harnessed at the societal level to contribute to the transformation.(Co-researcher)

Friction and resistance are described as positive courage, especially when experienced together. However, handling conversations about tension and friction requires facilitation, which, according to the reflections, may imply experience or trained ability.

Identify the tensions and take advantage of them. The tensions are golden nuggets. It is the tensions that are the movement. To bring about the change, we need to step outside the box and create tension and, above all, become better at listening. (Co-researcher)

Gathering around users’ needs and the fact that something positive is created out of this is fundamental in most stories. A problem shown through the reflections is the lack of everyday contexts or a forum where the transformation is on the agenda. In particular, questions concerning preventive work are needed.

### Emerging characteristics for a living arena

1. A neutral, illuminated and in-between space where people can come together.
2. A space that opens for different and new perspectives.
3. A possibility to decouple traditional and formal structures.

## Learning for emergence

Frequently recurring in the reflections is the need for change management, including following up, systematic learning and knowledge building. A more flexible or smooth organization is desired, and that working methods, that are proven successful, are quickly and systematically shared in the organization. However, almost no stories emerge where this is the case; instead, they are isolated actions based on local enthusiasts. Something that is also highlighted is the lack of reflection between the levels in the ecosystem—for example, concerning questions that need further exploration, or which insights truly influence decision-making—but also the time for reflection.

A gap between management and the operational level […] two groupings have a different view of how well they integrate each other into the work that is going on […] A new way of working between “new levels” is deeply rooted and is therefore regularly postponed […] even the simplest of things can be difficult to get hold of, experienced as difficult to prioritize—for example, when it is outside our daily/usual tasks and in a managerial role. (Co-researcher)

What I miss is having the time and space to make use of learning by doing, because most of the time it is putting out fires and fast pucks and then it becomes more ad hoc. (Co-researcher)

Learning in complexity seems to be dependent on actively listening and taking multiple perspectives—opening up to data. However, listening and being in other realities take time and can be difficult to achieve, and the reflections indicate that there can be fear that we hear things we do not want to hear, leading to a problem that seems to concern the absence of following up in general, which compromises the skill of listening.

How well the implementation process goes will partly depend on how well we can follow up the data but also the exchange of experience with those who do the work, those who collaborate within the municipality and region to create better understanding and preparation for those new at the job (Co-researcher)

Emergence in terms of prototyping and experimentation in the transformation—that we should be able to “pretend” before we make changes—has been a subject of reflection, saying that we can act as colleagues but are employed in different parts of organizations. The reflections indicate that we should dare to try or visualize something new for exploratory purposes and not for the purpose of verifying something that is meant to be implemented. This is especially true concerning areas or activities that may be norm breaking, but also in cases where a working hypothesis is believed to exist. Examples of cutting-edge phenomena such as sharing a budget across traditional borders are one thing; in contrast, simply collaborating across borders with the aim only of creating new relationships that may have an impact later (without knowing what that might be) may be something else.

Work operationally with solutions and strategically with structures that favor the transformation. Dare to work agilely in the sense that we dare to take steps even if we do not have all the solutions/the whole picture ready. Dare, aware that we will have to revise, start over, etc. (Co-researcher)

The co-researchers' reflections indicate that many good things are happening, but there are limitations to what is being structured and documented for learning. They note that the documentation that is produced does not become a visible whole, which would provide transparency and opportunities to act upon. Furthermore, in contrast to emergence, activities and projects that could be a part of the transformation must be articulated in advance, as well as what the result should be (top management expectations). This tends also to characterize the roles, functions or perspectives that are added to the work. An example is insufficient competence in analyzing and assessing resources to determine the right specific interventions. The same structures (roles, steering groups, methods, etc.) that are used to optimize and make everyday work more efficient also seem to be applied to what is more unknown and unexplored. Overall, a situation is described where you continue to do what you are doing but not what you should be doing.

Currently, we distinguish between three types of implementations: assignment, activity, and project. Projects are based on (the organization's) project model and are the traditional approach, with directives, plans and delivery […] it will be extremely difficult to carry out a shift to a more person-centered and integrated care with that mindset. (Co-researcher)

The study explores the potential of using SSM for gathering information (stories and experiences) via the people who are in the ecosystem 24/7. The co-researchers highlight a need for such complementary data gathering, using both quantitative (numbers, statistics) and more qualitative data (stories, events) to drive decisions. The experiences of employees, patients and relatives should be systematically considered in the same way as more traditional financial reports.

Difficult to get management to be sufficiently familiar with ongoing development work, which leads to fast and efficient processes during and as part of closure […] which leads to an experience of sprawl and repetition (the same question comes up several times and in parallel cooperation forums), with little responsibility to drive or distribute for increased coordination, consensus, or dialogue about the next step. Instead, the county/region [management] usually says thanks for the information and adds it to the documents while employees wonder what happens now? (Co-researcher)

### Emerging characteristics for learning in transformation

1. A need for systematic individual and collective reflection, documented.
2. An ability to use data and information.
3. A balance between thinking and doing.
4. An increased ability to listen in the organization.

## Leading for emergence

Leading emerging transformation requires active participation followed by a curious mindset for both formal and informal management roles. Management environments are credible when they are characterized by genuine teamwork: we do what others do, not what others tell us to do. The construct components are currently described as closed doors and closed rooms, where knowledge is perceived not as a deeper insight into needs or questions but rather as an assigned title. With that, the translation and interpretation that come with a change could risk ending up in rooms separated from one another, with the consequence of having less space to reinterpret or translate correctly. The definitions and the way they are formulated are based on the strongest logic. In this culture, the logics also shift with the different fields they pass through, such as politics, officials, professions and/or society.

[…] we opt out of the development potential in Nära Vård in favor of existing structures, hierarchies, and/or resources. Add to that, if the title manager is not included in the role description, a number of collaboration forums are automatically closed for knowledge acquisition/knowledge transfer, thus ensuring that knowledge does not flow throughout the system and across organizational boundaries as equal parties in a natural and uncontrived way where we strengthen networks and relationships. (Co-researcher)

Furthermore, there seems to be a partition between thinkers and doers. Roles in the system can be simply described as the manager, developer, creator and innovator, and employees can be described as objects, executors and administrators. In this view of resources, frustration is created around who is actually selected or has the opportunity to participate in development work.

We refuse to conduct surgery without a surgeon, we don't put people to sleep without anesthesia skills, we refuse to build houses without carpenters. To be able to prepare hospital food we use chefs … But we seem to be able to order and start improvement works without the blink of an eye and without securing the right skills for that very special mission. (Co-researcher)

Some of the reflections also highlight the need for ownership to be taken in change or transformation. The problem seems to be related to the fact that responsibility for specific issues is inadequate at different levels and that there is a lack of interaction and cocreation between levels. However, there are also signs that active actions from management create a feeling of “now it is happening for real”.

[…] there is also enthusiasm established among individuals who see an opportunity in development, which is positive and good, but managers need to become active enablers who go first and show the way and sometimes go both behind and alongside their employees with support and encouragement. (Co-researcher)

Acting as system management at all levels is described as necessary. What that actually means is not clear. However, the importance of being able to act more dynamically and meet new structures that may also be required to reach new goals or effects is evident. According to the stories received, the interaction in the management environment is quite invisible. Managers mostly take information back home and think about what it means for them instead of asking what this means for the collective or the ecosystem.

[…] it strikes me that there is no system management at any level. All management levels are minute operational—there is a lack of people NOW. We need to hunt down substitutes for the next work shift! And so everyone runs without thinking [ …]. There is no tradition of planned and preventive work, of bringing in facts and analyzing. (Co-researcher)

In other cases, it is mentioned that capacity, often described as being absent from the system, does actually exist—perhaps not at all times and in the right location, but knowledge and abilities exist in the system as a whole. However, it is unclear what type of capacity and resources can and should be distributed differently. No signals have been captured that could clarify a way, system or role/function to handle this issue.

Collaboration and shared responsibility are team games. They will never work when people/roles have their own (partially competing or corresponding) agendas […] we should be aware that it takes people who work precisely with these strategic issues—tasks that currently (due to resource savings?) often land with the managers […] (Co-researcher)

### Emerging characteristics for leading in transformation

1. A need for a resilient and adaptive organization.
2. A change in leadership view—leading each other instead of leading others.
3. A change in how resources (people) are defined.

## Using scientific social media to facilitate adaptive space and the core processes of transformation

In relation to previous research, the results of this study highlight many possibilities of facilitating the emergence of learning and quality in complexity by building relational structures that enable a new kind of adaptive space (Uhl-Bien and Arena, 2018). In the study, scientific social media became a critical addition to traditional forms and relations when it comes to facilitating people to explore, exchange and debate ideas more freely in connection to the Nära Vård transformation. It also clearly shifted the role of the facilitator from traditionally holding space in the room, or online, during specific real-time meetings and workshops, toward being a dialogue partner in a continuous asynchronous flow and network of reflections and dialogues with and between the many co-researchers.

In this case, the social media platform, along with collective reflection and analysis, can also be understood as supporting facilitation of the three core processes of transformation, as proposed within dialogic OD (Bushe and Marshak, 2016b). Through facilitation in the four loops, Table 4 connects the different processes with the three themes of action possibilities. Emergence, as pushing the system for transformation, is interpreted through the three themes: new spaces (living arenas), learning and leading, according to a new order that is needed for Nära Vård to happen. The subthemes can then be interpreted as generative images (Bushe and Marshak, 2016b), used as triggers for continuous dialogues and future exploration, showing alternative ways of acting in transformation and complexity—for example, how collective spaces should be understood, or how to systematically collect information and experiences. The change of story, or narrative (Bushe and Marshak, 2016b), is made possible by the collective sharing of experiences from members being in the system, reflecting together and the tension and possibilities they bring to the adaptive space. Stories from the co-researchers are to be found in the material and the overall reflection and co-analyzing process.

**Table 4:** Connecting the result to the core processes of organizational change in dialogic OD as highlighted by Bushe and Marshak (2016b) in relation to emergence coming through dialogue in a complex adaptive space (Uhl-Bien and Arena, 2018)

Enabling for a complex system to collectively sense, see and transform itself –In an adaptive space							
Emergence	A living arena			Learning		Leading	
Generative	Collective space	Participation	Catalysers	Basic needs	Systematics	Culture	Adaption
Narrative	A neutral, illuminated and in-between space for togetherness A space that opens up for different and new perspectives A possibility to decouple traditional and formal structures			A need for systematic individual and collective reflection – documented An ability to use data and information A balance between thinking and doing An increased ability to listen to the organization		A need for a resilient and adaptive organization A change in leadership view – leading each other instead of leading others A change in how resources (people) are defined	
Facilitation	Reflecting on the pressures from Nära Vård (Loop 1) Reflecting on insights (Loop 2) Reflecting on tensions and potential conflicts (Loop 3) Reflecting on hypotheses (Loop 4)						

**Source(s):** Authors’ own work

## Discussion and conclusion

In relation to the purpose of exploring needs and practices to facilitate the emergence of learning and quality in complexity with the support of scientific social media, the findings demonstrate and emphasize the value of creating a specific kind of space, a living arena, where people, and more specifically different perspectives, can meet to share, understand and allow learning and quality emerge. This could be understood as creating the conditions needed for interaction between people in relation to Stacey’s (2005) theories about complex change. In this study, it also became increasingly obvious that people in transformation struggle with the unknown and how to make things sufficiently concrete, indicating the importance of having more knowledge about complexity and transformational processes, as mentioned by Nuño-Solinís (2017) and Rognes *et al.* (2020). However, aligned to Uhl-Bien (2021), the tensions found in the reported reflections—for example, around organizing and logics in governance—bring a dynamic for emergence to happen. It is not necessarily more knowledge that is demanded, but rather a space to safely address the tensions and package them for organizational learning, giving organizational information to probe, sense and respond in an accurate way (Snowden and Boone, 2007).

Given the process of following and bringing in perspectives from people in the system in this way, we see how this could be described as digital listening, as highlighted by Pounsford *et al.* (2023). This is necessary, as employees are central to solving organizational problems. However, listening to employees is clearly linked to engagement, which is also emphasized in our results. The way of following members of the organization, with systematic reflection being documented and analyzed together, is also an opportunity to unfold multiplicity and complexity, providing space for uncertainty (Hersted *et al.*, 2020). What we find especially interesting is how this way of using action-oriented reflections and communicating with members of the organization returns to the theoretical perspectives of Scharmer (2018, p. 63), where we can help close some loops “between enactment of systems on a behavioral level and its source on the level of awareness and thought”. The use of SSM also relates to GMB (Gerritsen *et al*., 2020) and the potential to combine design, system and complexity thinking as elaborated by Teo *et al*. (2023).

This study has presented results that are aligned to parameters found in studies on leading during big change (Bevan and Fairman, 2017), indicating that another view and acting from a leadership perspective is necessary if transformation is to happen. We identify a leadership perspective needed for transformation, which relates to what Hiller *et al.* (2006) described as leadership that can be enacted collectively and informally by team members, as also seen in teal organizations according to Laloux (2020) and Weick’s (1995) seminal work on sensemaking and its importance in times of uncertainty and complexity.

In conclusion*,* this study had identified a triad with three themes of action possibilities for facilitating the emergence of learning and quality with the support of SSM. Three themes facilitate adaptive space and the core processes for transformation by (1) creating a living arena for emergence; (2) learning for emergence and (3) leading for emergence in transformation.

## Theoretical and practical implications

The science around systems thinking and complexity (Snowden and Boone, 2007; Stacey and Griffin, 2006; Uhl-Bien and Arena, 2018) involves many theories; more rarely does it involve practical ways of how to bring different perspectives to the table or the same room. With our research, we believe that we have presented an opportunity to do this in almost real time, but also in a way that builds a bridge from the theory to practical dilemmas, by using an SSM platform (Lackéus, 2014) and methodology from designed action sampling (Lackéus and Sävetun, 2023). Having the opportunity to share experiences, learning and stories this way was met with interest by the participants. As action researchers, we see the potential in times when resources are scarce, but we need to find ways of learning and transforming on the scale of complex ecosystems.

Although it may be too soon to say in relation to how we need to develop measurements and ways to follow changes in transformation and complexity (Rognes *et al*., 2020), this way of following members of organizations challenges the traditional concepts of evaluating and following up quality. Those terms represent a more reactive and retrospective approach, missing out on the opportunities to act during change. Therefore, we propose the concept of quality emergence, based on van Kemenade and Hardjono’s (2019) work on a new paradigm for quality emergence. Quality emergence sees quality as dynamic and constantly changing with the interaction between people and organizations. Being leaders in this system demands close following of the system—that is, relating to our methodological approach, following the members, users and their interconnections —what we in Swedish would call *medföljning* (co-following).

This study also gives a glimpse of how both to involve and to bring different perspectives together to get useful insights from complex change almost in real time. Combining theoretical exploration and practical action-oriented action in this way could give organizations an opportunity to explore the interactions between people and what consequences that may have for quality.

Practically, in relation to research on health systems transformation (Teo *et al.*, 2023) organizations could use SSM for seeing the system, understanding the system and working with the system.
